# MicroRNA in lung cancer

**DOI:** 10.1038/sj.bjc.6605901

**Published:** 2010-09-21

**Authors:** P-Y Lin, S-L Yu, P-C Yang

**Affiliations:** 1 Institute of Biomedical Sciences, Academia Sinica, Taipei, Taiwan; 2 Department of Internal Medicine, National Taiwan University Hospital, No. 7, Chung-Shan South Road, Taipei 100, Taiwan; 3 Department of Clinical and Laboratory Sciences and Medical Biotechnology, National Taiwan University, Taipei, Taiwan; 4 Division of Genomic Medicine, Research Center for Medical Excellence, National Taiwan University, Taipei, Taiwan

**Keywords:** lung cancer, microRNA, oncogene, tumour suppressor, risk stratification, personalised medicine

## Abstract

MicroRNAs (miRNAs) are small non-protein-coding RNAs that function as endogenous negative gene regulators. Dysfunctions of miRNAs are frequently found in malignancies, including lung cancer. In this review, we summarise the current understanding of miRNAs in lung cancer tumourigenesis, and highlight their potential in overcoming drug resistance, abetting histological sub-classification techniques, and serving as biomarkers for lung cancer risk stratification and outcome prediction.

Lung cancer is the leading cause of cancer mortality worldwide, and 80% of lung cancers are non-small cell lung cancers (NSCLCs) (Jemal *et al*, 2009). Despite improvements in early diagnosis made possible by emerging technologies and newly developed chemo/targeted therapies that improve treatment responses, the overall 5-year survival for NSCLC patients remains low (15%) and the recurrence rate is high, even in early-stage groups (Miller, 2005). The poor prognosis is due to late disease presentation, tumour heterogeneities within histological subtypes, and our relatively limited understanding of tumour biology. Emerging targeted therapies directed against specific cellular alterations require precise sub-classification of NSCLCs that is beyond the capabilities of standard histopathological diagnostic techniques. However, knowledge accumulated through genomic medicine creates the possibility of unravelling the remaining mysteries of lung cancer oncogenesis, and opens the door to molecular classification and risk stratification based on gene expression profiles and microRNA (miRNA) signatures.

MicroRNAs are small non-coding, endogenous, single-stranded RNAs that regulate gene expression (Bartel, 2004). Mature miRNAs and Argonaute (Ago) proteins form the RNA-induced silencing complex (RISC), which mediates post-transcriptional gene silencing through induction of messenger RNA (mRNA) degradation or translational inhibition (Liu *et al*, 2004; Pillai *et al*, 2004). Target mRNA specification is determined by sequence complementarity between the seed sequence of an individual miRNA and the target mRNAs (Baek *et al*, 2008; Eulalio *et al*, 2008; Selbach *et al*, 2008; Bartel, 2009). Recent proteomic studies have revealed a broad spectrum of targets for each individual miRNA (Baek *et al*, 2008; Selbach *et al*, 2008). By regulating gene expression at the post-transcriptional level, miRNAs profoundly influence a wide variety of pathways, and their greatest impact is on developmental and oncogenic pathways (Lee *et al*, 1993; Wightman *et al*, 1993; He *et al*, 2005, 2007; Johnson *et al*, 2005; Lu *et al*, 2005; Calin and Croce, 2006).

Half of all miRNA genes are found within or near chromosomal fragile sites, common breakpoints, or minimal regions of loss-of-heterozygosity or amplification (Calin *et al*, 2004). Accumulating evidence shows that miRNAs are grossly dysregulated in human cancers, including NSCLC, and may serve as oncogenes or tumour suppressors (Croce, 2009). Recent studies have shown that not only can miRNAs be used to sub-classify NSCLCs (Bishop *et al*, 2010) but specific miRNA profiles may also predict prognosis and disease recurrence in early-stage NSCLCs (Yanaihara *et al*, 2006; Yu *et al*, 2008; Raponi *et al*, 2009; Seike *et al*, 2009; Patnaik *et al*, 2010).

In this review, we briefly describe the biogenesis of miRNAs, their roles in lung cancer pathogenesis, and their potential use in NSCLC subgroup classification, risk stratification, and therapy. The stratification of lung cancer based on miRNA information is illustrated in Figure 1.

## MicroRNA biogenesis

MicroRNA genes are evolutionarily conserved and are located within the introns or exons of protein-coding genes, as well as in intergenic areas (Rodriguez *et al*, 2004). Canonically, miRNA genes are transcribed by RNA polymerase II or III into kilobase-long primary miRNA transcripts (pri-miRNAs). Pri-miRNAs are next cleaved into ∼70 nucleotide-long precursor miRNAs (pre-miRNAs) by the nuclear microprocessor complex formed by the RNase III Drosha and DiGeorge syndrome critical region gene 8 (DGCR8). The average human pre-miRNA contains a 33-base-pair hairpin stem, a terminal loop, and two single-stranded flanking regions upstream and downstream of the hairpin. Pre-miRNAs are next transported by the exportin-5/Ran GTPase complex into the cytoplasm, where miRNAs undergo maturation (Lee *et al*, 2003, 2004; Yi *et al*, 2003; Denli *et al*, 2004). In the cytoplasm, pre-miRNAs are cleaved by RNase III Dicer into an ∼22 nucleotide-long miRNA duplex and are unwound by helicase. The passenger strand is degraded, and the selected guide strand together with Ago protein activates RISC, resulting in mRNA degradation or translational inhibition, depending on the percentage of sequence complementarity between the miRNA 5′-seed and mRNA 3′-UTR element (Hammond *et al*, 2000; Diederichs and Haber, 2007). Alternatively, pre-miRNAs are derived directly from size-matched introns. These so-called ‘mitrons’ skip the Drosha–DGCR8 processing step and are spliced out of their host genes. These lariats are debranched, refolded into the stem-loop structure of typical pre-miRNAs, and then enter the canonical pathway (Okamura *et al*, 2007; Ruby *et al*, 2007).

A recent study by Suzuki *et al* demonstrated that p53 interacts with the Drosha microprocessor complex through DEAD-box RNA helicase p68 (DDX5) and facilitates the processing of pri-miRNAs into pre-miRNAs. This study describes the p53-mediated post-transcriptional maturation of miRNAs, linking the core tumour suppressor p53 to the miRNA biogenesis pathway (Suzuki *et al*, 2009). These findings may provide an explanation for the widespread miRNA downregulation observed in human cancers, in which p53 is often dysfunctional (Lu *et al*, 2005).

## Defects in the miRNA biogenesis pathway and lung cancer

Drosha, DGCR8, and Dicer are the three best-established regulators of miRNA processing. Defects in the miRNA biogenesis machinery may be closely related to oncogenesis. Deletion of Dicer abrogates the production of mature miRNAs (Bernstein *et al*, 2003), and conditional deletion of Dicer1 enhances lung tumour development in a K-Ras-induced lung cancer mouse model (Kumar *et al*, 2007). In 2005, Karube *et al* (2005) reported that reduced Dicer expression levels were correlated with poor survival in a cohort of 67 surgically resected NSCLC patients. This correlation has been confirmed in an ovarian cancer, three breast cancers, and a lung cancer cohort in which high Dicer and Drosha mRNA expression is associated with better overall and disease-free survival (Merritt *et al*, 2008).

## MicroRNAs function as tumour suppressors or oncogenes in lung cancer

### *Let-7* miRNAs as tumour suppressors

*Let-7* was first identified in *Caenorhabditis elegans* as a regulator of the timing of cell fate determination (Reinhart *et al*, 2000). In *C. elegans* with mutant *let-7*, stem cells fail to exit the cell cycle and differentiate, but continue to divide, a hallmark of cancer cells (Reinhart *et al*, 2000). In humans, the *let-7* family is a cluster of miRNAs whose genes map to different chromosomal regions that are frequently deleted in lung cancer (Calin *et al*, 2004). Cell studies have shown that *let-7* miRNA overexpression in the A549 cell line inhibits cell growth and reduces cell-cycle progression (Johnson *et al*, 2007). In mouse NSCLC xenograft and orthotopic models, ectopic *let-7g* expression reduces tumour burden (Kumar *et al*, 2008), and intranasal *let-7* administration represses lung adenocarcinoma (AD) formation (Esquela-Kerscher *et al*, 2008). Furthermore, reduced *let-7* gene expression in NSCLC patients is correlated with poor prognosis (Takamizawa *et al*, 2004; Yanaihara *et al*, 2006), and a single nucleotide polymorphism in *let-7* complementary site 6 of the *K-RAS* mRNA 3′-UTR is significantly associated with increased risk for NSCLC among moderate smokers (Chin *et al*, 2008). Collectively, these observations suggest a role for *let-7* family miRNAs as tumour suppressors. In addition, *let-7* miRNAs negatively regulate multiple oncogenes, including the *RAS* (Johnson *et al*, 2005), *MYC* (Kumar *et al*, 2007), and *HMGA2* (Lee and Dutta, 2007), and cell-cycle progression regulators, such as *CDC25A*, *CDK6*, and cyclin D2 (Johnson *et al*, 2007). Although a corresponding knockout mouse would be invaluable for in-depth studies of *let-7* tumour suppressor functions, multiple copies of *let-7* in the genome make generating such a model somewhat problematic (Calin *et al*, 2004).

## All members of the *miR-17-92* cluster and *miR-31* are oncogenes

All members of the *miR-17-29* cluster (*miR-17, miR-18a, miR-19a, miR-20a, miR-19b-1, miR-92-1*) are oncogenes that reside in 13q31.3 (He *et al*, 2005). These miRNAs cooperate with c-Myc to accelerate tumour development and help promote tumour neovascularisation (O’Donnell *et al*, 2005; Dews *et al*, 2006). The *miR-17-92* cluster is overexpressed in small-cell lung cancer (Hayashita *et al*, 2005). Ebi *et al* (2009) have confirmed this relationship and reported the association of *miR-17-92* overexpression with RB inactivation. Their results suggest that this miRNA cluster may be a potential therapeutic target in lung cancer.

*miR-31* is another example of an miRNA with oncogenic properties (termed an oncomir) in lung cancer. As reported by Liu *et al*, knockdown of *miR-31* represses lung cancer cell clonal growth and *in vivo* tumourigenicity. Their data show that *miR-31* functions as an oncomir by directly repressing the tumour suppressors LATS2 and PPP2R2A (Liu *et al*, 2010). This miR-31/LATS2/PPP2R2A pathway constitutes a new growth regulator in lung cancer.

## MicroRNAs and conventional chemotherapy for NSCLCs

The selection for chemotherapy-resistant cells is often observed in platinum-based chemotherapy, currently the main regimen in NSCLC treatment (Seve and Dumontet, 2005), and is a key cause of chemotherapeutic failure. A recent *in vitro* study by Galluzzi *et al* (2010) showed that *miR-630* inhibits p53-regulated pro-apoptotic signalling pathways that are specifically induced by cisplatin and carboplatin. This is one example demonstrating that the role of miRNAs may involve chemo-sensitivity/-resistance determination and suggesting the possibility that manipulating miRNAs may be potentially useful to modulate the cancer chemoresistance.

## miRNAs and targeted therapies

Epidermal growth factor receptor (EGFR) signalling and *EGFR* mutations have been a major focus of lung cancer studies conducted during the past 5 years. Epidermal growth factor receptor is one of the most common proto-oncogenes in lung cancer. Leucine-to-arginine substitution at position 858 (L858R) and deletion mutants in exon 19 constitute 90% of lung cancer-specific EGFR-activating mutations. These mutations induce lung AD in a mouse model and confer hypersensitivity to EGFR–tyrosine kinase inhibitors (EGFR–TKIs) in humans (Rosell *et al*, 2009).

Several recent studies have uncovered a relationship between the EGFR signalling pathway and miRNAs. Weiss *et al* (2008) showed that *miR-128b* is a direct regulator of *EGFR*. *miR-128b* loss-of-heterozygosity is frequently found in NSCLC patients and is positively correlated with clinical response and survival after gefitinib treatment. In addition, Cho *et al* (2009) showed that restoration of the tumour suppressor *miR-145* inhibits cancer cell growth in lung AD patients with EGFR-activating mutations. Furthermore, *miR-7*, an miRNA frequently downregulated in lung cancer, has been shown to suppress *EGFR* and *Raf1* mRNA expression. It also attenuates the activation of Akt and ERK, two key players in the EGFR signalling pathway, suggesting that *miR-7* negatively regulates the EGFR pathway (Webster *et al*, 2009). Finally, *miR-21* is upregulated under conditions in which EGFR signalling is activated, especially in the context of EGFR-activating mutations, and is suggested to be related to lung carcinogenesis in never smokers (Seike *et al*, 2009). Growing evidence from miRNA studies may help clarify the role of the EGFR network in lung cancer oncogenesis and provide a clue to solve EGFR—TKI resistance problems.

The Apo2L/tumour necrosis factor (TNF)-related apoptosis-inducing ligand (TRAIL) is a member of the TNF family known to induce apoptosis in a variety of cancers (Schaefer *et al*, 2007). Treatment with TRAIL induces programmed cell death in cancer cells. However, a significant proportion of cancers are resistant to TRAIL-induced apoptosis through different mechanisms. Garofalo *et al* (2009) showed that *miR-221* and *miR-222* contribute to lung cancer resistance to TRAIL therapy by downregulating PTEN and TIMP3 tumour suppressors. These observations hint at the extent to which a greater knowledge of miRNAs might bring a deeper understanding of drug-resistance mechanisms, and suggest a future role for miRNAs as a solution to the drug resistance problem.

## MicroRNAs in NSCLC histological differentiation, risk stratification, and outcome prediction

### Histological differentiation

With the emergence of targeted therapies directed against specific molecular events or entities, accurate classification of tumours into AD and squamous cell carcinoma (SCC) becomes a necessity. However, this can be challenging, especially in cases in which biopsy/aspirate specimens are small or tumours are poorly differentiated. *miR-205* is reported to be a useful marker for differentiating SCC from non-SCC NSCLCs, with a sensitivity of 96% and specificity of 90%, even in small biopsies from poorly differentiated tumours (Lebanony *et al*, 2009; Bishop *et al*, 2010). Landi *et al* (2010) also reported a five-miRNA signature (*miR-25, miR-34c-5p, miR-191, let-7e,* and *miR-34a*) that accurately differentiated SCC from AD, and the lower expression level of this signature correlated with poor overall survival among SCC patients. This is a further step beyond the traditional protein markers used in immunohistochemical diagnosis of equivocal cases. It is anticipated that miRNA markers will ultimately also be found for AD.

### Risk stratification and outcome prediction

Risk stratification and prognosis assessment have become a major concern in the era of personalised medicine. Gene expression profiling has reached a plateau in this regard (Garber *et al*, 2001; Shedden *et al*, 2008), although recent miRNA studies show great promise (Yanaihara *et al*, 2006; Yu *et al*, 2008; Raponi *et al*, 2009).

Yanaihara *et al* (2006) reported that high *miR-155* and low *miR-let7a-2* expression correlated with poor overall survival in lung AD patients. Our group also identified a five-miRNA signature (miR-137, miR-372, miR-182*, *miR-221*, *and let-7a*) that correlated with disease-free survival in a cohort of 122 NSCLC patients (Yu *et al*, 2008). In addition, Raponi *et al.* reported that *miR-146b* is a robust predictor of overall survival in SCC. High *miR-146* expression correlated with a poor overall survival in SCC patients and the same trend was also observed in the expression level of *miR-155* among the same group of patients (Raponi *et al*, 2009). The pathway prediction of *miR-146b*-targeted genes revealed a significant overlap of biological pathways in their previously reported 50-gene expression signature (Beer *et al*, 2002; Raponi *et al*, 2009). Patnaik *et al* (2010) also defined an miRNA signature that predicted post-operative recurrence of stage I NSCLCs. It is clearly evident from these studies that, as is the case with gene expression profiling, miRNA signatures suggested by different groups are almost non-overlapping. This could be because of the different experimental platforms used (qRT–PCR versus different miRNA array systems), batch effects inherent in the microarray experiment itself, and potential ethnic differences in the study populations, as observed in the EGFR mutation story. More detailed studies are needed to clarify these issues. In addition, new modalities, such as next-generation sequencing, may provide tools to enhance the prospects of miRNA research.

A recent study by Hu *et al* (2010) reported a four-miRNA signature (*miR-486, miR-30d, miR-1*, *and miR-499*) that predicted survival of stage I to IIIa NSCLCs. Unlike all previous studies, their miRNAs were identified in serum using a Solexa platform (Nanjing Medical University, Nanjing University and Jiangsu Cancer Hospital, Nanjing, China). Although this represents a relative non-invasive approach, questions remain as to the representativeness of serum miRNA profiles in solid cancers, given that this approach fails to identify miRNAs commonly found in lung cancer tissues.

## Perspectives

Numerous miRNAs are dysregulated in cancers, and a single miRNA can have multiple targets involved in different oncogenic pathways. Accumulating evidence also suggests a role for miRNAs in fighting drug resistance. These properties make miRNAs attractive targets in cancer therapy. However, the fact that one miRNA may target hundreds of mRNAs deserves special consideration, insofar as it implies the possibility of unpredictable side effects, even if a specific miRNA is effectively targeted. A greater understanding of miRNA biology and the development of suitable delivery systems are required to translate these basic research results into clinical practice. In addition, miRNAs may abet the histological characterisation of NSCLC differentiation, especially in cases in which biopsy/aspiration specimens are inadequate or tumours are poorly differentiated. This is especially important when targeted therapy is the treatment of choice. Risk stratification and drug-response prediction are the central elements of personalised medicine. During recent years, research has focused mainly on gene expression profiling and a number of important studies have been published. As gene expression profiling reaches a plateau and begins to face limitations, miRNA signature is a rising star that may provide new resolutions to old problems. It is difficult to say whether miRNA signature is superior or inferior to gene expression profiling, with respect to risk stratification and outcome prediction. What is clear, however, is that the more we understand cancer biology, the more likely we are to translate these laboratory-oriented studies into clinical practice. Finally, miRNAs are much more stable in serum and plasma than in mRNAs, raising the exciting prospect that miRNAs might be used as non-invasive biomarkers for disease monitoring and histological classification under specific circumstances. Going forward, insights gained from miRNA studies may open a new era in lung cancer treatments, providing improved patient selection for targeted agents, and forming the basis for the development of novel therapeutics and/or early disease biomarkers.

## Figures and Tables

**Figure 1:**
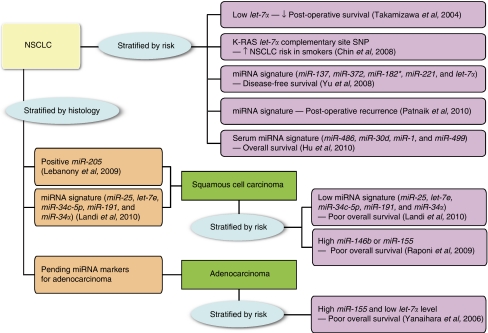
Stratification of lung cancer based on microRNA information. NSCLC, non-small-cell lung cancer.
